# Spontaneous Pathologic Splenic Rupture in a Patient with *Plasmodium falciparum* Infection, First Case Reported in Israel

**DOI:** 10.3390/idr12030022

**Published:** 2020-12-08

**Authors:** Yves Weinberg, Arie Feldman, Daniel J. Jakobson, Joseph Mishal

**Affiliations:** 1 Internal Medicine B Department, Barzilai University Medical Center, Ashkelon 7830604, Israel; aryef@bmc.gov.il (A.F.); mishael@bmc.gov.il (J.M.); 2 Faculty of Health Sciences, Ben-Gurion University of the Negev, Beer-Sheba 8410501, Israel; danielj@bmc.gov.il; 3 Department of Oral and Maxillofacial, Barzilai University Medical Center, Hahistadrut 2, Ashkelon 7830604, Israel; 4 Intensive Care Unit, Barzilai University Medical Center, Ashkelon 7830604, Israel

**Keywords:** malaria, spleen rupture, *Plasmodium falciparum*

## Abstract

Travelers exposed to malaria may develop severe disease and complications. A less well-known complication is spontaneous pathologic splenic rupture, which is still under-reported and has never been reported in Israel. In this paper, we report a 23 years old healthy young man presenting in the emergency department, two weeks after coming back from Sierra Leone, with intermittent fever, mild tachycardia and mild left upper quadrant abdominal pain. The patient was diagnosed with *Plasmodium falciparum* infection and developed rapidly after hospital admission spleen rupture. He was managed conservatively at first but ultimately underwent splenectomy after being hemodynamically unstable. In the recovery period, the patient developed acute respiratory distress syndrome and was reintubated. A high level of suspicion is recommended in every malaria patient presenting with left upper quadrant abdominal pain, even if minimal. Ultrasonography availability in the internal medicine department may be a critical diagnostic tool, especially in non-endemic areas.

## 1. Introduction

Malaria is a mosquito-borne infection affecting humans. The disease is transmitted by a single cell microorganism from the *Plasmodium* group, *Plasmodium falciparum* (*P. falciparum*) having the largest burden followed by *Plasmodium vivax* (*P. Vivax*) [1]. *P. falciparum* predominates in Sub-Saharan Africa, New Guinea and Hispaniola (Haiti and the Dominican Republic) [2].

In 2017, the World Health Organization (WHO) reported 216 million cases of malaria worldwide and 445,000 malaria death cases (death peak in 2004—1.24 million cases) [3]. The prevalence of the disease declined by 37% from 2000 to 2015. Approximately 90% of cases of death occurred in Africa.

Endemic area populations are at lower risks for severe disease, except for young children, pregnant women and immunocompromised patients (including a-splenic patients). Travelers, on the other hand, being exposed to the parasite for the first time, are at very higher risks for severe disease [4,5].

Many of the clinical findings of severe malaria are the result of the parasitized (and nonparasitized) red blood cells (RBC) adhering to small blood vessels (“cytoadherence”), causing small infarcts, capillary leakage and organ dysfunction [6]. Complications include cerebral malaria, metabolic acidosis, hypoglycemia, acute respiratory distress syndrome (ARDS), renal failure with hemoglobinuria, hepatic failure and hematologic complications (coagulopathy with or without disseminated intravascular coagulation (DIC), severe anemia and massive intravascular hemolysis).

Another, less well known, life-threatening complication of malaria is spontaneous pathologic splenic rupture [7]. This complication is rare but probably under-reported. In this paper, we report a patient with *Plasmodium falciparum* infection who developed within a few hours spontaneous splenic rupture. He was managed at first with conservative management but required emergent splenectomy due to hemodynamic deterioration.

## 2. Description

A 23-year-old healthy young man arrived at the emergency room with a one-day history of intermittent fever with chills and sweating.

He reported that two weeks before the onset of fever, he returned from a volunteer mission in Sierra Leone, West Africa. He received malaria preventive treatment with mefloquine before and during his stay but was discontinued on the day he left the endemic area, despite the usual recommendations to continue treatment for another four weeks. He reported that he slept, during his stay, in a hut without a net and was stung by mosquitoes several times.

On physical examination, he had very mild upper left quadrant abdominal pain with no distention, tenderness or rebound. He reported vomiting once.

At presentation, his body temperature was 36.9 °C, he had mild tachycardia 103 beats/min, and blood pressure was 99/69 mm/Hg.

In blood tests, he had thrombocytopenia, C-reactive protein (CRP) was elevated, and he had hyponatremia disturbance in liver function. The data are shown in Table 1.

Chest X-ray was normal. The electrocardiogram (ECG) was normal except for tipped T-waves on the V3, V4 and V5 leads.

He was admitted to the internal medicine B department, malaria was suspected. Thick and thin blood smear preparations were performed and confirmed parasitemia. *P. falciparum* was identified; the parasitemia level was 0.1–0.2%. In addition, *P. falciparum* antigen was detected on the CareStart malaria Rapydtest kit and confirmed later by PCR.

At this point, the patient was stable, had no fever and seemed to be feeling well. Anti-malarial treatment with PO artemether/lumefantrine 20 mg/120 mg was started. Four tablets were initially administered initially and four tablets more later on the same day. In addition, IV doxycycline, 100 mg/day, was administered.

A few hours later, his body temperature raised to 39 °C, and he reported acute abdominal pain; the abdomen was tender on palpation. Bedside abdominal ultrasound (US) revealed free peritoneal fluids. At this stage, he was moderately hypotensive, HB was 10 g/dL. Abdominal computed tomography (CT) scan discovered a grade III spleen rupture and hemoperitoneum.

**Figure 1:**
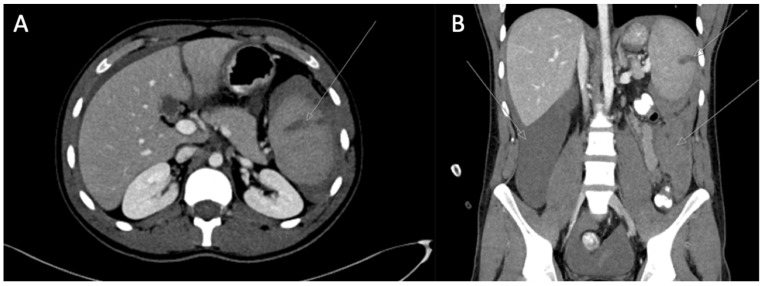
Abdomen CT scan. (**A**) Axial view; (**B**) coronal view. Arrows point at grade III spleen rupture and hemoperitoneum.

Spontaneous pathologic splenic rupture related to *Plasmodium falciparum* infection was diagnosed. This case is depicted in Figure 1.

## 3. Treatment

On consultation with the general surgery department, conservative management was proposed, and the patient was transferred to the intensive care unit.

While on monitoring, five units of platelets and one packed red blood cells were administered. He was stable overnight, but then suddenly, the systolic blood pressure decreased to 60 mmHg, and his pulse rate was 140 beats/min. The patient was immediately transferred to the operating theatre for laparotomy. Total splenectomy was performed, and the patient was transferred back to the intensive care unit. On the same day, PO artemether/lumefantrine 20 mg/120 mg was discontinued and was replaced with IV quinidine 1843 mg, at a rate of 0.02 mg/kg/min.

## 4. Outcomes

Extubation was performed 6 h after surgery. After being able to reinitiate PO treatment, the patient was administered another two doses of artemether/lumefantrine 20 mg/120 mg, four tabs each time.

**Figure 2:**
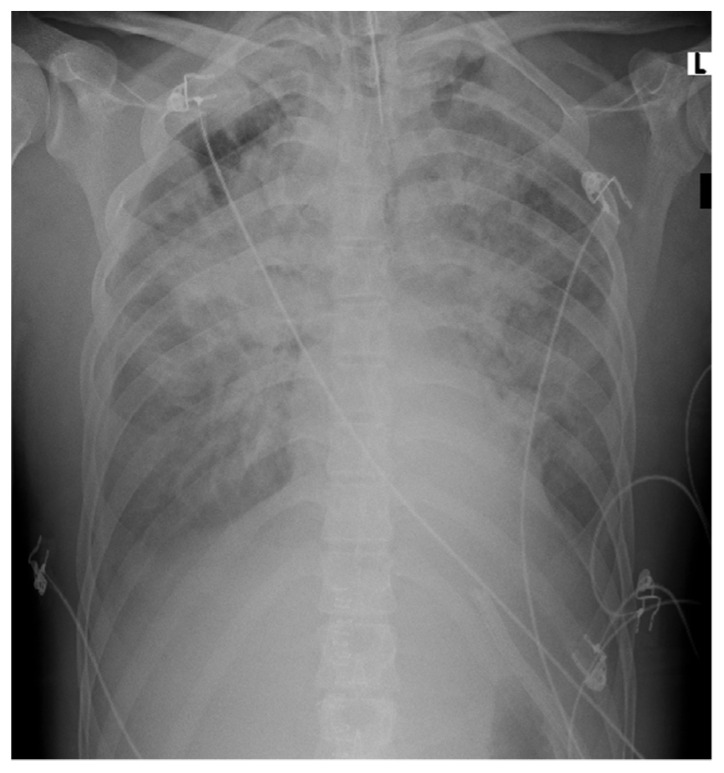
Patient chest X-ray. (**L**) left side of the patient.

Later on, the patient progressively became respiratorily distressed, and 26 h after extubation, he needed invasive respiratory support because of severe hypoxemia. Chest X-ray showed diffuse infiltrations, and bilateral pleural effusions were seen; ARDS was diagnosed. Turbid pleural effusion was drained and diagnosed as transudate according to Light’s criteria. This case is depicted in Figure 2.

The day later, the patient’s status improved, and he was extubated. At this stage, no malaria Parasites were observed on blood smears.

After splenectomy, platelet levels raised above one million, and enoxaparin sodium treatment was started.

After discharge, the patient was referred to as capsulated agents vaccination.

## 5. Discussion

Malaria should be suspected in the setting of symptoms consistent with malaria and positive malarial diagnostic test. Treatment should be administered as soon as possible in order to prevent complications. In this case, anti-malarial treatment with four tablets of artemether/lumefantrine 20 mg/120 mg was started. However, after the patient was intubated, he was administered IV quinidine instead of IV artesunate because IV artesunate was not available in our institution.

Rupture of the spleen is relatively common following significant blunt abdominal injury. Malaria is considered the most frequent cause of spontaneous pathological rupture of the spleen worldwide [7]. Other cases may be associated with invasive procedures (colonoscopy being the most reported) [8], with infiltrative or inflammatory pathologies, e.g., infectious diseases (malaria being the most reported), hematology diseases (non-Hodgkin’s lymphoma is the most reported), rheumatologic diseases (ex. Systemic lupus erythematosus (SLE), Wegener granulomatosis) or Amyloidosis. It may also be associated with a previously diagnosed splenic or adjacent physical abnormality, pregnancy-related causes and non-hematologic neoplastic causes. It may be related to medications (anticoagulant being the most reported) and internal trauma (ex. Cough, vomiting, and seizures). Cases of pure spontaneous splenic rupture, without any pathology, are much rarer and have been reported in the literature only in 35 papers until 2012 [8].

Splenic architecture is altered by malaria, and various mechanisms have been proposed in the process of splenic rupture: increased intra-splenic tension caused by cellular hyperplasia and engorgement, compression by the abdominal musculature during physiological activities such as sneezing, coughing, defection, and vascular occlusion caused by reticular endothelial hyperplasia with results in thrombosis and infarction [9].

The diagnosis of spleen rupture in the setting of malaria should be made as soon as possible in order to improve mortality and morbidity. A 22% mortality rate was reported in 2009 [7]. The death rate among travelers was higher than in other patients (38% versus 10%). If a patient with malaria complains of left upper quadrant abdominal pain, pleuritic lower chest and/or enlarging tender splenomegaly (before or even during anti-malarial treatment), splenic hematoma or splenic infarct should be suspected [10]. The patient should be closely monitored and managed accordingly to avoid further life-threatening complications in case of spleen rupture, especially for residents in non-endemic areas.

In this case, our patient presented in the emergency room with an intermittent fever two weeks after he came back from Sierra Leone. He had thrombocytopenia. In physical examination, he had mild tachycardia, and he had minimal left upper quadrant pain. All these symptoms should have raised suspicion for imminent spleen rupture related to malaria. The diagnosis was made only a few hours later when the symptoms worsened and confirmed with bedside US.

Point-of-care ultrasonography in the internal medicine department has been proved to be a useful diagnostic tool in many aspects of department activities; it is also highly efficient for diagnosing hemoperitoneum and increasing suspicion for spleen rupture [11]. In this case, it has been performed almost immediately after acute symptoms started, and it has been critical in order to save the patient’s life.

Treatment of splenic rupture remains debated. As of today, there are no specific guidelines for spontaneous pathologic spleen rupture, and it should follow the same principles as in trauma cases. Spleen conserving measures should be performed whenever possible, especially in patients with potential further exposure to malaria [7]. Splenectomy is the standard treatment for patients with uncontrollable bleeding, for patients hemodynamically unstable and for patients with recurrent spleen bleeding. Spleen suture should be considered whenever possible. In well-equipped facilities, embolization of the splenic artery may be performed.

In this case, a more aggressive approach, with immediate splenectomy, could have prevented blood transfusions and maybe further ARDS complication, even if this complication may be directly related to malaria.

## 6. Conclusions

Spontaneous pathologic splenic rupture related to *Plasmodium falciparum* infection is a rare, not known enough, life-threatening condition. It could have devastating consequences. Treatment providers should have a high level of suspicion in every malaria patient presenting with left upper quadrant abdominal pain or enlarging tender splenomegaly, even if the pain is minimal. These patients should be closely monitored, and US availability in the internal medicine department may be critical in order to save their lives.

Appropriate treatment for this condition is still debated. A conservative approach is recommended whenever possible, but, in our case, a more aggressive approach with total splenectomy was needed to prevent further complications.

## Figures and Tables

**Table 1:** Blood tests.

Blood Test	Result	Unit	Normal Value
White blood cells (WBC)	5.150	10^9^/L	4.000–10.000
Hemoglobin (HB)	15.8	g/dL	13–17
Platelets (PLT)	47	10^9^/μL	150–450
C-reactive protein (CRP)	98	mg/L	<0.6–5.00
Sodium (Na)	131	mmol/L	136–145
Alanine aminotransferase (ALT)	58	U/L	10–41
Aspartate aminotransferase (AST)	56	U/L	10–40
Lactate dehydrogenase (LDH)	683	U/L	240–480
Alkaline phosphatase (alkphos)	91	U/L	40–130
Gamma-glutamyl transferase (GGT)	99	U/L	8–60
